# Effect of Metal Ions on Melanin – Local Anaesthetic Drug Complexes

**DOI:** 10.1155/S1565363303000098

**Published:** 2003

**Authors:** Ewa Buszman, Bożena Betlej, Dorota Wrześniok, Bożena Radwańska-Wala

**Affiliations:** Medical University of Silesia School of Pharmacy Department of Pharmaceutical Chemistry Jagiellońska str. 4 Sosnowiec 41-200 Poland

## Abstract

The affinity of melanin biopolymers for metal ions, drugs and other organic compounds is an important factor in the etiology of toxic retinopathy, hiperpigmentation, otic lesions and irreversible extrapyramidal disorders. The aim of the presented work was to examine the interaction of local anaesthetic drugs used in ophthalmology with model DOPA-melanin in the presence of metal ions. It has been demonstrated that the analyzed drugs form complexes with melanin biopolymer. Based on the .values of association constants,, the following order of drugs affinity to melanin was found: tetracaine > procaine >> bupivacaine > lidocaine. It has also been shown that Cu^2+^ and Zn^2+^ ions administered to DOPA-melanin before complexing with drugs decrease the total amount of local anaesthetics bound to melanin. The blocking of some active centers in melanin molecules by metal ions, which potentially exist in living systems, may change the clinical therapeutic efficiency of the analyzed local anaesthetic drugs.


### Effect of Metal Ions on Melanin – Local Anaesthetic Drug Complexes

Ewa Buszman*, Bożena Betlej, Dorota Wrześniok and Bożena Radwańska-Wala

*Medical University of Silesia, School of Pharmacy,*
*Department of Pharmaceutical Chemistry,*
*Jagiellońska str. 4, 41-200 Sosnowiec, Poland*

(Received: November 8, 2002; Accepted: December 30, 2002)

### ABSTRACT

The affinity of melanin biopolymers for metal ions, drugs and other organic compounds is an important factor in the etiology of toxic retinopathy, hiperpigmentation, otic lesions and irreversible extrapyramidal disorders. The aim of the presented work was to examine the interaction of local anaesthetic drugs used in ophthalmology with model DOPA-melanin in the presence of metal ions. It has been demonstrated that the analyzed drugs form complexes with melanin biopolymer. Based on the values of association constants, the following order of drugs affinity to melanin was found: tetracaine > procaine >> bupivacaine > lidocaine. It has also been shown that $Cu^{2+}$ and $Zn^{2+}$ ions administered to DOPA-melanin before complexing with drugs decrease the total amount of local anaesthetics bound to melanin. The blocking of some active centers in melanin molecules by metal ions, which potentially exist in living systems, may change the clinical therapeutic efficiency of the analyzed local anaesthetic drugs.

**Key words:** melanin, local anaesthetics, metal ion-melanin complexes, drug-melanin complexes

### INTRODUCTION

Melanins are biopolymers synthetized in melanosomes of the melanocyte /1,2,3/. The pigmentation system is genetically controlled at multiple levels, including migration of melanocytes during embryogenesis

---

* *Corresponding author: Ewa Buszman, Medical University of Silesia, School of Pharmacy, Department of Pharmaceutical Chemistry, Jagiellońska str. 4, 41-200 Sosnowiec, Poland, Tel. 48-32-292-55-44, Fax: 48-32-266-78-60, E-mail: ebuszman@slam.katowice.pl and melanin synthesis at cellular, subcellular, and enzymatic levels. The precursors of melanins are tyrosine, DOPA and dopamine, which give eumelanin after oxidation, subsequent cyclization and polymerization. Detailed molecular studies of melanin synthesis have shown that it is a very complex process involving multiple enzymes /4/. Of all enzymes involved in the process, only tyrosinase is essential for the synthesis of melanins /5/. The level and activities of melanogenic enzymes tyrosinase, TRP-1 and TRP-2 are regulated by the MSH cell surface receptor and the melanosomal P-protein – the two most obvious candidate genes influencing variation in pigmentation phenotype /6,7/. Melanins occur in various tissues including skin, hair, hair follicles, retinal pigment epithelium, choroid and iris of the eye, stria vascularis of the cochlea and planum semilunatum of the ampullae of the ear and substantia nigra of the brain /8/. This pigment is found in the nerve cells of the brain of larger animal species including man, in macrophages within the superficial cervical lymph nodes /3/ and as component of human eosinophils /9/.

It is well known that a number of drugs and other chemicals are accumulated and retained for long periods in pigmented tissues due to melanin affinity. The heterogeneity of substances with melanin affinity is large: various drugs of different categories (psychotropic drugs, drugs for rheumatoid arthritis and malaria, antineoplastic drugs, aminoglycoside antibiotics, local anaesthetics), herbicydes, dyes, alkaloids and metal ions /10,11,12,13,14,15/. The accumulation of certain drugs in pigmented tissues, due to melanin affinity, is possibly the most pronounced retention mechanism of the body. This accumulation is generally believed to be the important factor in the etiology of toxic retinopathy, hyperpigmentation of the skin, hair bleaching, irreversible extrapyramidal disorders, some ocular and inner ear lesions /8,16,17,18,19/.

It is also known that metal ions, which potentially exist in living systems, bind to melanin biopolymers /20/ and may affect the drug-melanin interaction. The melanin-metal ions binding has been ascribed to a cation exchange activity of melanin, which in turn may be related to the presence of free carboxyl groups in the melanin biopolymer. It was demonstrated that pigmented cells contain a rather high concentration of metal ions as compared with non-melanized homologous tissues /21,22,23/. Furthermore, oxidative transformations of melanin precursors (DOPA, dopamine, adrenaline) are catalyzed by $Cu^{2+}$ and $Cu^{2+}$-containing enzymes /24/. It has been postulated that the affinity of melanins for metal ions can be an important factor in the pathogenesis of some lesions, e.g. long-time exposure to manganese may cause chronic extrapyramidal disorders due to metal accumulation on neuromelanin /25/.

Increasing use of intraocular local anaesthetics for ophthalmic surgery calls for a better understanding of the mechanisms of various undesirable drugs effects. Long-term administration of local anaesthetics to the eye has been associated with retarded healing and pitting and sloughing of the corneal epithelium and predisposition of the eye to inadvertent injury /26,27,28/.

The aim of this study was to examine the interaction of local anaesthetic drugs used in ophthalmology (lidocaine and bupivacaine) with model DOPA-melanin in the presence of metal ions ($Cu^{2+}$ and $Zn^{2+}$ ).

### EXPERIMENTAL

### Melanin synthesis

Model synthetic melanin was obtained by oxidative polymerization of L-3,4-dihydroxyphenylalanine (L-DOPA from Sigma) solution (1mg/ml) in 0.067 M phosphate buffer (pH 8.0) for 48h, according to the Binns method /29/.

### Metal ions-melanin complex formation

Dry DOPA-melanin samples of 300mg each were mixed with 300ml of bidistilled water containing $1 \cdot 10^{-3}$M concentration of $Cu^{2+}$ ($CuCl_2 \cdot 2H_2O$, PPH POCH, Poland) or $Zn^{2+}$ ($ZnCl_2$, Merck, Germany). The mixtures were incubated at room temperature for 24h and then filtered. The amounts of copper and zinc bound to melanin were determined by the use of atomic absorption spectrophotometer type AAS 3 (Carl Zeiss, Jena). The final metal ions-DOPA-melanin complexes contained 0.43μmol $Cu^{2+}$/mg melanin and 0.30μmol $Zn^{2+}$/mg melanin.

### Drug-melanin complex formation

Binding of lidocaine (lidocaine hydrochloride, Polfa, Poland) and bupivacaine (bupivacaine hydrochloride, Molteni Farmaceutici, Italy) to melanin was performed in 0.067M phosphate buffer at pH 7.0. Drug-melanin complexes were obtained as follows: 5mg of melanin or metal ion-melanin complexes were placed in plastic test-tubes, where drug solutions in phosphate buffer were added to a final volume of 5ml. The initial concentration of drugs ranged from $1 \cdot 10^{-4}$M to $1 \cdot 10^{-3}$M. Control samples contained 5mg of melanin and 5ml of buffer without drug. All samples were incubated for 24 h at room temperature, and then filtered.

### Analysis of drug binding to melanin

The concentrations of drugs remaining in each filtrate after incubation with melanin were determined spectrophotometrically. All spectrophotometric measurements were made using the UV-VIS spectrophotometer Jasco model V-530. The amounts of drug bound to melanin, calculated as the difference between the initial amount of drug administered to melanin and the amount of unbound drug (in filtrate after incubation) were expressed in μmoles of bound drug per 1mg melanin. The complex formation efficiency (in %) was also determined as the ratio of drug (μmole) bound to melanin to the total amount of drug (μmole) added to melanin, multiplied by 100. A qualitative analysis of drug-melanin interactions was performed using the Scatchard plot of the experimental data according to Kalbitzer and Stehlik /30/. The number of binding sites (n) and the values of association constant (K) were calculated.

### RESULTS

The effect of metal ions on local anaesthetics-melanin complex formation efficiency is presented in Table 1. The affinity of lidocaine and bupivacaine to melanin and metal ion-melanin complexes was analyzed as described in the Experimental section, whereas the results for procaine and tetracaine complexes are known from previous studies /31/. As shown in Table 1, the two-fold increase of initial drug concentration (from $5 \cdot 10^{-4}$M to $1 \cdot 10^{-3}$M) results in the decrease of percentage of drug bound to melanin or metal ion-melanin complex. Simultaneously, the increase of the absolute amount of local anaesthetics bound to melanin (in

**Table 1**

Binding of local anaesthetics to DOPA-melanin as well as to metal ions- melanin complexes[a].

| Analyzed drug | Initial drug concentration [M] | % Binding to melanin | | |
|---|---|---|---|---|
| | | melanin | $Cu^{2+}$-melanin | $Zn^{2+}$-melanin |
| Lidocaine | $5 \cdot 10^{-4}$ | 33.5 | 20.6 | 13.5 |
| | $1 \cdot 10^{-3}$ | 24.1 | 14.9 | 10.3 |
| Bupivacaine | $5 \cdot 10^{-4}$ | 22.1 | 15.1 | 10.4 |
| | $1 \cdot 10^{-3}$ | 18.6 | 9.0 | 6.5 |
| Procaine[b] | $5 \cdot 10^{-4}$ | 74.0 | 63.6 | 65.4 |
| | $1 \cdot 10^{-3}$ | 60.4 | 53.4 | 54.9 |
| Tetracaine[b] | $5 \cdot 10^{-4}$ | 67.4 | 65.6 | 59.3 |
| | $1 \cdot 10^{-3}$ | 52.6 | 42.2 | 39.4 |

[a]5mg melanin were incubated with 5ml of drug solutions for 24h at pH 7.0.

[b]Results from previous studies in the lab /31/

µmoles per mg melanin) with the increase of initial drug concentration has been demonstrated (Fig. 1A). $Cu^{2+}$ and $Zn^{2+}$ ions fixed by melanin before complexing with drug modify the local anaesthetics binding ability. The decrease of the amount of drug bound to melanin in the presence of metal ions was observed, especially in the presence of $Zn^{2+}$, as compared with drug-melanin complexes obtained in the absence of metals.

The experimental data were also analyzed by constructing the Scatchard plots to determine the binding sites and the number of relevant binding classes. For lidocaine-melanin and bupivacaine-melanin complexes the Scatchard plots are linear (Fig. 1B), indicating that one class of binding sites participates in the formation of these complexes. For procaine-melanin and tetracaine-melanin complexes the Scatchard plots were found to be curvilinear /31/, which indicates that more than one binding class was involved. The calculated binding parameters for the interaction of local anaesthetics with melanin and metal ion-melanin complexes are shown in Table 2. The values of association constant for lidocaine-melanin and bupivacaine-melanin complexes ($K \sim 10^3 M^{-1}$) demonstrate that mainly weakly reacting sites exist in these complexes. For procaine-melanin and tetracaine-melanin interactions strong binding sites with the association constant $K_1 \sim 10^5 M^{-1}$ and weak binding sites with $K_2 \sim 10^3 M^{-1}$ were stated. In the presence of $Cu^{2+}$ and $Zn^{2+}$ ions an approximately two-fold

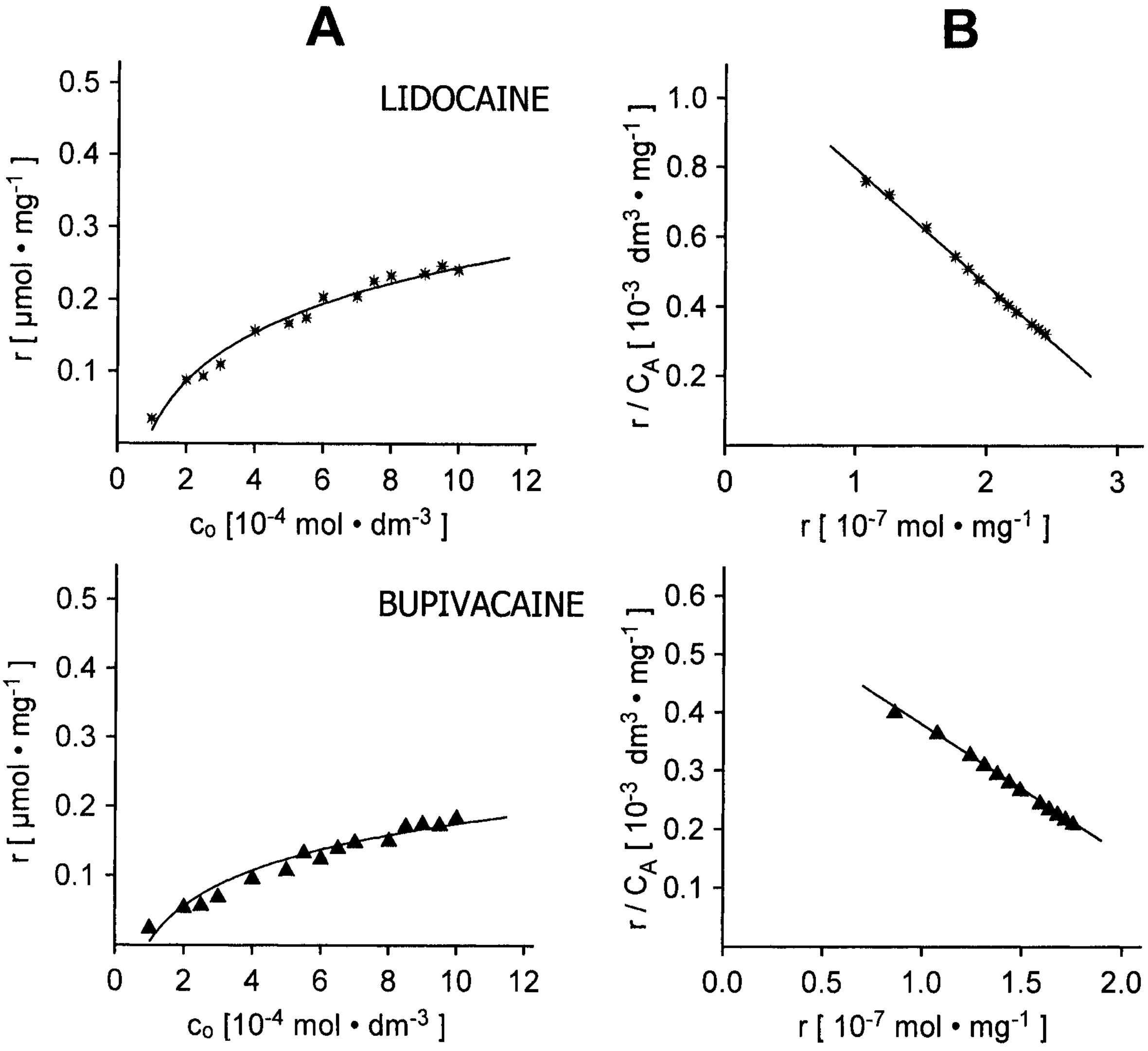

**Fig. 1:** Binding isotherms (**A**) and Scatchard plots (**B**) for lidocaine and bupivacaine complexes with DOPA-melanin. r – amount of drug bound to melanin, $C_0$ – initial drug concentration, $C_A$ – concentration of unbound drug decrease of the number of binding sites (n) was observed for lidocaine and bupivacaine interaction with melanin. For procaine and tetracaine complexes with melanin the decrease of total binding capacity ($n_{tot}$) in the presence of metal ions was also demonstrated.

The presented results indicate that $Cu^{2+}$ and $Zn^{2+}$ ions complexed with melanin modify the binding ability of local anaesthetic drugs by blocking some active centers in the melanin molecules.

**Table 2**

Binding parameters for local anaesthetics-melanin and local anaesthetics-(metal ions-melanin) complexes.

| Analyzed complex | Lidocaine | | Bupivacaine | | Procaine[a] | | Tetracaine[a] | |
|---|---|---|---|---|---|---|---|---|
| | Association constants K /$M^{-1}$/ | Number of binding sites n /μmol drug/ mg melanin/ | Association constants K /$M^{-1}$/ | Number of binding sites n /μmol drug/ mg melanin/ | Association constants K /$M^{-1}$/ | Number of binding sites n /μmol drug/ mg melanin/ | Association constants K /$M^{-1}$/ | Number of binding sites n /μmol drug/ mg melanin/ |
| Drug-melanin | $K = 3.32 \cdot 10^3$ | n = 0.340 | $K = 2.30 \cdot 10^3$ | n = 0.267 | $K_1 = 2.05 \cdot 10^5$<br>$K_2 = 4.20 \cdot 10^3$ | $n_1 = 0.320$<br>$n_2 = 0.402$<br>$n_{tot} = 0.722$ | $K_1 = 3.61 \cdot 10^5$<br>$K_2 = 4.43 \cdot 10^3$ | $n_1 = 0.239$<br>$n_2 = 0.410$<br>$n_{tot} = 0.649$ |
| Drug-($Cu^{2+}$-melanin) | $K = 1.73 \cdot 10^3$ | n = 0.183 | $K = 2.00 \cdot 10^3$ | n = 0.103 | $K_1 = 3.10 \cdot 10^5$<br>$K_2 = 4.28 \cdot 10^3$ | $n_1 = 0.234$<br>$n_2 = 0.318$<br>$n_{tot} = 0.552$ | $K_1 = 0.68 \cdot 10^5$<br>$K_2 = 2.48 \cdot 10^3$ | $n_1 = 0,206$<br>$n_2 = 0.354$<br>$n_{tot} = 0.560$ |
| Drug-($Zn^{2+}$-melanin) | $K = 2.62 \cdot 10^3$ | n = 0.218 | $K = 2.34 \cdot 10^3$ | n = 0.139 | $K_1 = 1.91 \cdot 10^5$<br>$K_2 = 3.58 \cdot 10^3$ | $n_1 = 0.230$<br>$n_2 = 0.349$<br>$n_{tot} = 0.579$ | $K_1 = 1.32 \cdot 10^5$<br>$K_2 = 3.05 \cdot 10^3$ | $n_1 = 0.224$<br>$n_2 = 0.359$<br>$n_{tot} = 0.583$ |

[a] Results from previous studies in the lab /31/

### DISCUSSION

The eye is located externally in the body and is vulnerable to injury and exposure to toxic environmental insults. Despite its small mass, it carries out biochemical processes which require an avid and diverse blood supply. This results in this organ being second only to the liver in showing manifestations of drug toxicity especially from compounds present in the systemic circulation /18/.

Optical measurements of the pigments of the retinal pigment epithelium (RPE) and choroid have shown that choroidal melanin was always greater than that in the RPE. Simultaneously, it was found that the content of both choroidal and RPE melanin showed a trend of decreasing with aging /32/. Prota *et al.* /33/ have stated that in human irides both eumelanin and pheomelanin are present, which indicates that differences in stromal pigmentation may be due not only to the quantity, but also to the nature of melanin pigment. It has also been suggested that the differences between human brown and blue eyes in their melanin content may have relevance to the pharmacokinetics of drugs that bind to melanin. This would mean that the larger amounts of melanin would decrease the initial levels of the drugs and would increase the drug levels after prolonged periods /34/.

Although the biology of the pigment granules has been neglected in the past, they do seem to be involved in many important functions, such as protection from oxidative stress, detoxification of peroxides, and binding of metal ions and drugs, and, therefore, serve as a versatile partner of the retinal pigment epithelium (RPE) cell. New findings show that the melanin granules are connected to the lysosomal degradation pathway. Most of these functions are not yet understood, but it was demonstrated that the deficit of melanin pigment is associated with age-related macula degeneration, the leading cause of blindness /35/.

At present, many drugs are known to be markedly accumulated and retained for a considerable time by pigmented tissues and that the retention of these compounds is proportional to the degree of melanin pigmentation /10/. The ability of melanins to bind different drugs and transition metal ions is probably of the greatest biological importance /10,20,25,36/.

Many previous studies have focused on the ability of a compound to bind to melanin *in vitro* as a measure of its affinity for melanin-containing structures *in vivo*. The experimental approach often entails the isolation of melanin from biologic sources or the preparation of synthetic melanin from its precursors /37/. Synthetic melanins prepared enzymatically or chemically from L-DOPA contain more carboxyl groups than natural melanins /12/. Furthermore, one can assume that the ion-exchange, redox and free radical properties established in synthetic melanin are also relevant for natural melanins /38/. Both natural and synthetic melanin polymers have been used in binding studies of ligands and no significant differences in the affinity were observed /12,39/. The earlier experiments with synthetic melanin have shown that metal ions had the same order of affinity for synthetic melanin as for eye-melanin /20/. This suggests that the protein moiety of the melanin plays a minor role in the binding of metal ions. However, a high affinity of metal ions for melanin in vitro does not necessarily mean that a high uptake occurs also in vivo. Melanin is an intracellular constituent, and transport barriers at the cell membranes may prevent ions from reaching the melanin in vivo /20/.

More complete analysis of the metal ions-melanin interaction was performed earlier in our laboratory /36,40,41/. It was shown that two classes of independent binding sites participate in the interaction of $Fe^{3+}$,

$Co^{2+}$, $Ni^{2+}$, $Cu^{2+}$, $Zn^{2+}$, $Cd^{2+}$, $Cr^{3+}$ and $Sn^{2+}$ ions with DOPA-melanin, whereas for $Mn^{2+}$ ions only one class of binding sites was found. Sarna *et al.* /22/ and Froncisz *et al.* /42/ have shown that, depending on pH and the metal ion concentration, metals form various complexes with melanin biopolymer that involve different groups of the polymer and show different stabilities. Thus monodentate, bidentate and even tetradentate complexes of $Cu^{2+}$ ions with synthetic and natural melanin have been identified and a role of melanin carboxyl, phenolic hydroxyl, amine and imine groups has been discussed. It has been also reported that binding of metal ions to melanin is a time-dependent process /38,41/.

The effect of metal ions on drug binding ability to melanin was examined previously /40/. It has been demonstrated that in the interaction of chloroquine with synthetic DOPA-melanin two classes of independent binding sites must be implicated. Metal ions ($Cu^{2+}$, $Zn^{2+}$, $Mn^{2+}$, $Co^{2+}$, $Ni^{2+}$) fixed by melanin polymer before complexing with drug are the blocking agents, because only one class of binding sites has been found for chloroquine-(metal ion-melanin) complexes /43/. It has also been shown that metal ions incorporated into melanin during its synthesis modify the drug binding ability /44,45/.

The results presented in this study indicate that local anaesthetics used in ophthalmology form complexes with melanin biopolymer. Based on the values of association constants, the following order of drugs affinity to synthetic DOPA-melanin was found: tetracaine > procaine >> bupivacaine >lidocaine. It has also been demonstrated that $Cu^{2+}$ and $Zn^{2+}$ ions administered to DOPA-melanin before complexing with drugs decrease the total amount of local anaesthetics bound to melanin. The nature of the melanin–ligand interactions is still not well established but the existence of ionic bonds, Van der Waals forces, hydrophobic interactions and change-transfer reactions in drug-melanin complex formation has been suggested /10/. Taking into account that metal ions as well as the analyzed drugs exist in the form of cations at physiological pH, probably the same active centers in melanin polyanion are responsible for these ligands binding. The blocking of some active centers in melanin molecules by metal ions which potentially exist in living systems may change the clinical therapeutic efficiency of the analyzed local anaesthetic drugs.

### ACKNOWLEDGEMENTS

This work was financially supported by the Medical University of Silesia (Grant NN-4-287/02)

### REFERENCES

1. J. Lambert, G. Vancoillie and J.M. Naeyaert, *Cell Mol. Biol*, **45**, 905 (1999).
2. U. Mårs and B.S. Larsson, *Pigment Cell Res*, **12**, 266 (1999).
3. N.G. Lindquist, *Acta. Radiol. (Stockh)*, Suppl. **325**, 1 (1973).
4. V.J. Hearing and K. Tsukamoto, *FASEB J*, **5**, 2902 (1993).
5. J.P. Ortonne, *Br. J. Dermatol*, **146** (Suppl. 61), 7 (2002).
6. R.A. Sturm, R.D. Teasdale and N.F. Box, *Bioessays*, **20**, 712 (1998).
7. R.A. Sturm, N.F. Box and M. Ramsay, *Gene*, **277**, 49 (2001).
8. R.M.J. Ings, *Drug Metab. Rev*, **15**, 1183 (1984).

9. M.R. Okun, B. Donnellan, S.H. Pearson and L.M. Edelstein, *Lab. Invest*, **30**, 681 (1974).
10. B.S. Larsson, *Pigment Cell Res*, **6**, 127 (1993).
11. M.M. Salazar-Bookaman, I. Wainer and P.N. Patil, *J. Ocul. Pharmacol*, **10**, 217 (1994).
12. H. Ibrahim and A.F. Aubry, *Anal. Biochem*, **229**, 272 (1995) .
13. A. Radwańska, T. Frackowiak, H. Ibrahim, A.F. Aubry and R. Kaliszan,. *Biomed. Chromatogr*, **9**, 233 (1995).
14. D. Wrześniok, E. Buszman, J. Trzcionka and R. Różańska, *Ann. Acad. Med. Siles*, **40-41**, 63 (1999).
15. A. Surażyński, J. Pałka, D. Wrześniok, E. Buszman and P. Kaczmarczyk, *Eur. J. Pharmacol*, **419**, 139 (2001).
16. L. Lyttkens, B.S. Larsson, H. Göller, S. Englesson and J. Stahle, *Acta Otolaryngol*, **88**, 61 (1979).
17. S.A. Wästerström, *Scand. Audiol*, Suppl. **23**,1 (1984) .
18. P. Eves, L. Smith-Thomas, S. Hedley, M. Wagner, C. Balafa and S. Mac Neil, *Pigment Cell Res*, **12**, 22 (1999).
19. D. Wrześniok, E. Buszman, E. Karna, P. Nawrat and J. Pałka, *Eur. J. Pharmacol*, **446**, 7 (2002).
20. B.S. Larsson and H. Tjalve, *Acta Physiol. Scand*, **104**, 479 (1978).
21. A.A. Kochańska-Dziurowicz, T. Wilczok, L. Mosulishvili and N. Kharabadze, *Stud. Biophys*, **113**, 267 (1986).
22. T. Sarna , W. Froncisz and J.S. Hyde, *Arch. Biochem. Biophys*, **202**, 304 (1980).
23. H.M. Swartz, T. Sarna and L. Zecca, *Ann. Neurol*, Suppl. **32**, S69 (1992).
24. G. Prota, *Melanins and Melanogenesis*, Academic Press Inc. New York, 1992.
25. A. Lyden, B.S. Larsson and N.G. Lindquist, *Acta Pharmacol. Toxicol, (Copenh)*, **55**, 133 (1984).
26. A.J. Judge, K. Najafi, D.A. Lee and K.M. Miller, *Ophthalmology*, **104**, 1373 (1997).
27. C.L. Grosskreutz, W.R. Katowitz, E.E. Freeman and E.B. Dreyer, *Curr. Eye. Res*, **18**, 363 (1999).
28. M. Guzey, A. Satici, Z. Dogan and S. Karadede, *Ophthalmologica*, **216**, 113 (2002).
29. F. Binns, R.F. Chapman, N.C. Robson, G.A. Swan and A. Waggot, *J. Chem. Soc.* (C), 1128 (1970).
30. H.R. Kalbitzer and D. Stehlik, *Z. Naturforsch*, **34c**, 757 (1979).
31. E. Buszman, B. Betlej and D. Wrześniok, *Ann. Acad. Med. Siles*, **50-51**, 13 (2002) .
32. J.J. Weiter, F.C. Delori, G.L. Wing and K.A. Fitch, *Invest. Ophthalmol. Vis. Sci*, **27**,145 (1986).
33. G. Prota, D.N. Hu, M.R. Vincensi, S.A. McCormick and A. Napolitano, *Exp. Eye Res*, **67**, 293 (1998).
34. I.A. Menon, D.C. Wakeham, S.D. Persad, M. Avaria, G.E. Trope and P.K. Basu, *J. Ocul. Pharmacol*, **8**, 35 (1992).
35. U. Schraermeyer and K. Heimann, *Pigment Cell Res*, **12**, 219 (1999).
36. E. Buszman, B. Kwaśniak and A. Bogacz, *Stud. Biophys*, **125**, 143 (1988).
37. P.R. Raghavan, P.A. Zane and S.L. Tripp, *Experientia*, **46**, 77 (1990).
38. T. Sarna, *J. Photochem. Photobiol. B: Biol*, **12**, 215 (1992).
39. M.M. Salazar, T. Sokoloski and P.N. Patil, *Fed. Proc*, 37, 2403 (1978).
40. E. Buszman, *Binding of Drugs to Melanin Biopolymers in the Presence of Metal Ions*, Hab. Thesis, Medical University of Silesia, Katowice, 1994.
41. E. Buszman, B. Kwaśniak, Z. Dzierżewicz, and T. Wilczok, *Stud. Biophys*, **122**, 147 (1987).
42. W. Froncisz, T. Sarna and J.S. Hyde, *Arch. Biochem. Biophys*, **202**, 289 (1980).

43. E. Buszman, B. Kwaśniak and T. Wilczok, *Proc. IIIrd Symp. on Inorg. Biochem. and Mol. Biophys*, Wrocław, 163, 1991.

44. E. Buszman, Z. Dzierżewicz, B. Kwaśniak and T. Wilczok, *Stud. Biophys*, **122**, 139 (1987).

45. E. Buszman, B. Kwaśniak and T. Wilczok, *Curr. Top. Biophys*, **16**, 81 (1992).

